# Alcohol use and sickness absence due to all causes and mental- or musculoskeletal disorders: a nationally representative study

**DOI:** 10.1186/s12889-018-5059-8

**Published:** 2018-01-17

**Authors:** Leena Kaila-Kangas, Aki Koskinen, Päivi Leino-Arjas, Marianna Virtanen, Tommi Härkänen, Tea Lallukka

**Affiliations:** 1 Work ability and working careers, Finnish Institute of Occupational Health, PL 40, 00251 Helsinki, Finland; 2 Creating Solutions, Statistics and Health Economics Team, Finnish Institute of Occupational Health, Helsinki, Finland; 3 Department of Health, Functional Capacity and Welfare, National Institute for Health and Welfare, Helsinki, Finland; 4 Department of Public Health, University of Helsinki, Helsinki, Finland

**Keywords:** Abstinence, Alcohol use disorders, Mental disorders, Sickness absence, Work ability

## Abstract

**Background:**

Previous studies have not distinguished between different alcohol-use histories, which could have contributed to the current inconsistent evidence regarding the relationship between alcohol use and subsequent sickness absence. We thus examined alcohol use and subsequent diagnosis-specific sickness absence in groups with different levels of alcohol use, as well as in lifelong abstainers, former drinkers, and people with clinical alcohol use disorders.

**Methods:**

The data of the population-based Health 2000 Survey (BRIF8901) of 3666 Finns aged 30–55 were linked with national registers on medically certified sickness absences lasting for > 10 working days (long-term) for all causes (2000 − 2010) and for mental or musculoskeletal disorders (2004-2010), as well as with registers on pensions and death (2000-2010). Alcohol use was assessed by questionnaire. Chronic somatic diseases were evaluated at baseline in a clinical examination, and common mental and alcohol use disorders using the Composite International Diagnostic Interview (CIDI). Cox regression analyses were conducted with censoring for death and retirement from work.

**Results:**

During an average 10-year follow-up, 56.0% of the participants had at least one long-term sickness absence period. Compared with light drinkers, those having an alcohol use disorder had increased risk of all-cause sickness absence (HR = 1.27; 95% CI = 1.04 − 1.54) and sickness absence due to mental disorders (HR = 2.16; 95% CI = 1.39 − 3.35), when somatic and mental disorders as well as demographic, lifestyle-related and occupational factors at baseline were accounted for. Lifelong abstainers did not differ from light drinkers. Also high-volume drinking (HR = 1.52; 95% CI 1.03 − 2.25) and former drinking (HR = 1.57; 95% CI = 1.15 − 2.15) were associated with long-term sickness absence due to mental disorders. Alcohol use was not predictive of sickness absence due to musculoskeletal disorders.

**Conclusions:**

These results highlight the need to distinguish between former drinking and lifelong abstinence, as only former drinking was associated with sickness absence. Alcohol use disorder and high-volume drinking were strongly predictive of sickness absence due to mental disorders. Identifying people with excessive alcohol use e.g. in occupational health care, and mapping and supporting their mental health may help in preventing sickness absences.

## Background

Yearly costs of alcohol misuse in Finland have currently been about 1 thousand million euros to the public sector, via several kinds of disturbance and impairment, costs comprising about 1 % of the total gross domestic product [1]. The proportion of disability pension and sickness absence benefits was about a quarter of the total alcohol-related costs. Alcohol-related harms and costs are also considerable on the global level [2, 3].When a population is rapidly ageing, as in Finland, it is important to promote its health and work ability by minimizing factors that further reduce work participation.

A recent review [4] showed moderate evidence that alcohol consumption is associated with both short and longer sickness absence, but these associations did not systematically vary across the different categories of alcohol use. Some studies have found a linear association between average alcohol consumption and sickness absence at the population level [5–7], whereas others have found a U-shaped association, i.e. an increase of sickness absence among problem drinkers but also among abstainers [8–11]. Some studies have failed to find any association [12, 13]. Moreover, longitudinal population based studies with clinically verified alcohol use disorder are lacking, as are studies that present sickness absence according to diagnostic groups.

Our previous study [14] showed that abstinence did not predict disability retirement when former drinkers and lifelong abstainers were examined separately. Instead, former drinking and especially having a clinical alcohol use disorder (dependence and/or abuse) were strong determinants of disability retirement due to mental disorders. Alcohol dependence is usually a long-drawn condition, even though many sufferers periodically try to reduce or quit drinking and therefore probably move from one alcohol-use category to another. This may confound study results, and so we observed also participants with alcohol use disorder separately, regardless of their weekly use of alcohol.

We found only one study about the relationship between alcohol use and sickness absence due to musculoskeletal disorders [15]. The lack of studies may be due to scarce and conflicting study results concerning the impact of alcohol use on musculoskeletal disorders. Heavy alcohol consumption is considered to increase the risk of osteoporosis and bone fracture [16, 17] and have a detrimental effect on bone microarchitecture [18], but some studies have found that moderate alcohol consumption has a beneficial effect on bone density, with a lower risk of, for example, hip fracture [19]. As for widespread musculoskeletal pain, some studies of the normal population have found no association with alcohol use [20], although a U-shaped relationship has been reported among men [21].

The aim of this study was to examine the association of alcohol use with subsequent sickness absence lasting over 10 days. We analysed sickness absences due to any cause, as well as those due to musculoskeletal and mental disorders – the diagnostic groups that cause about one half of all nationally registered sickness absence periods [22]. We compared lifelong abstainers, former drinkers, moderate and high-volume drinkers, and people with alcohol use disorder, to light drinkers in a nationally representative sample of working aged Finns.

## Methods

### Procedure and participants

The baseline data were extracted from the nationally representative Health 2000 Survey, which was conducted between 2000 and 2001 in Finland [23], coordinated by the National Public Health Institute. The data collection comprised a number of structured questionnaires and interviews, laboratory and functional capacity tests, and a clinical examination by a physician. The participants signed their written informed consent also for future registry linkages. The study protocol was approved by the Ethics Committee for Epidemiology and Public Health for the hospital district of Helsinki and Uusimaa. The data collection process is described in detail elsewhere [23].

**Fig. 1:**
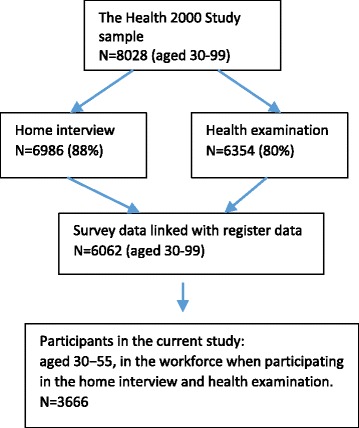
Flowchart of the study

Of the original sample of 8028 people aged 30 years or over (Fig. 1), 51 died before the data were gathered. Of the remaining 7977 people 6986 (88%) were interviewed and 6354 (80%) participated in a health examination [23]. To examine sickness absence, we restricted the follow-up to the participants, who were 30 − 55 years old at baseline, had participated in the clinical examination and home interview, filled in self-administrated questionnaires and did not die or retire during the follow-up (*N* = 3666). Because of the age restriction, all our participants had the possibility to be followed up until the official retirement age that was 65 years.

### Register data

Sickness absence data consisted of the starting and ending dates and medically certified diagnoses of compensated sickness absence periods per year, retrieved from the national registries of the Social Insurance Institution in Finland. Information comprising sickness absence due to any diagnosis was complete from 2000 to 2010, while that on mental and musculoskeletal disorders was available from 2004 to 2010. Diagnoses before 2004 were based on the systematic sampling and were recorded for only those whose birthdate was the 18th or 28th. In Finland, a prerequisite of sickness allowance is a waiting period, which covers the day of onset of work incapacity and the following nine working days. Thus the sickness absence data comprised all compensated sickness absence periods that lasted more than 10 working days. All non-retired Finnish citizens aged 16-67 are eligible for a daily sickness insurance allowance to compensate for loss of earning for a maximum of 300 working days because of a particular physician-diagnosed disease. Compensation for lost income is based on annual earnings of the individual. If it is anticipated that the disability continues longer than this, the person must apply for either a temporary or permanent disability pension. The diagnoses of chronic illnesses in the national sickness absence register are classified according to The International Statistical Classification of Diseases and Related Health Problems, Tenth Revision (ICD-10) [24]. Here we used the following categorization of causes of sickness absence: any causes, musculoskeletal disorders (M00 − M99) and mental disorders (F00 − F99).

National registers of Finnish Centre for Pensions provided complete information on all retirement events and their main diagnoses. Data on deaths were obtained from Statistics Finland. All register data were linked to the Health 2000 data by each participant’s personal identification number that all Finns receive at birth [25]. We analysed the anonymized data without any identity codes.

### Measurement of alcohol use

The general frame for classification of alcohol use was developed in our previous study on the relationship between alcohol and disability pension [14], but here we applied lower portion limits for hazardous alcohol drinking, in line with current evidence-based national recommendations [26].

Using a self-administered questionnaire the participants were first inquired about their alcohol consumption during the past 12 months as follows 1) “I have been sober all my life (or have tasted alcohol beverages a maximum of 10 times at the most in my life)” (yes/no), 2) “I have consumed alcohol beverages since __ (year) but quit __ years ago”, and 3) “I have consumed alcohol beverages since __ (year) and still do”.

Participants who had never used alcohol were categorized as “*lifelong abstainers*” and those who had quit and reported no alcohol consumption during the past week as “*former drinkers*”. Participants who fulfilled the diagnostic criteria of alcohol dependence or alcohol abuse were categorized as having an *alcohol use disorder* regardless of their current level of alcohol intake. Those who did not meet the criteria of an alcohol use disorder, were classified into three groups according to the number of standard drinks per week: 1) *light*; 1 − 6 for men, 1 − 4 for women, 2) *moderate;* 7 − 22 for men, 5 − 11 for women, and 3) *high-volume* (hazardous); ≥ 23 for men and ≥12 for women.

Alcohol use was further assessed by two questions [27]: “How often have you consumed alcohol during the past 12 months?” and “On the days when you consumed alcohol, how much did you drink?” The frequency question had ten response alternatives. The beverage-specific (mild beverages/wine/spirits) amounts were also inquired and converted into standard drinks (a standard drink = 12 g of pure alcohol). The quantity-frequency method (QF) for each of the three beverage types was calculated by multiplying the weekly quantities by the weekly frequencies. These beverage-specific QFs were summed as a total QF measure. The proportion of missing data in alcohol-use was 1.8%.

The 12-month prevalence of *alcohol use disorder* was assessed using the computerized version of the Composite International Diagnostic Interview (CIDI) [28], carried out by healthcare workers trained for the interview by psychiatrists and physicians. The program uses operationalized criteria for diagnoses from the DSM-IV [29]. The diagnosis of alcohol use disorders include alcohol dependence and alcohol abuse. The criteria for alcohol dependence comprise increased tolerance, withdrawal, continued use despite problems, and impaired control. Alcohol abuse refers to conditions of obvious adverse consequences caused by alcohol use. Ninety (2.5%) participants did not take part in the CIDI interview, but most of these (88%) nonetheless answered the alcohol use questionnaire.

### Potential confounders

We selected the covariates on the basis of the literature on factors possibly confounding the association between alcohol use and sickness absence. The variables are presented in more detail elsewhere [23]. The home interview collected information on *gender, age (continuous), basic education (≤ 9 yrs. and > 9 yrs)* and *marital status (single, cohabiting)*. Depressive and anxiety disorders (12-month prevalence) were assessed using the computerized version of the Composite International Diagnostic Interview (CIDI) [28]. The participants were classified as having a *mental disorder* (yes/no) if they fulfilled the criteria for a depressive disorder, (i.e. major depressive disorder, dysthymic disorder) or anxiety disorder, (i.e. panic disorder with or without agoraphobia, generalized anxiety, social phobia not otherwise specified, or agoraphobia without panic disorder). In addition, the participants were categorized as having a *mental disorder* if a physician diagnosed one of the following: psychosis, depression or anxiety. *Musculoskeletal disorders* were diagnosed in the clinical examination on the basis of disease history, symptoms, and clinical findings. The participants were categorized as having a chronic disease if a physician diagnosed one of the following: cardiovascular-, respiratory- or neurological disease, diabetes, cancer, peptic ulcer or permanent injury. Weight and height were measured and the *body mass index* **(**BMI, kg/m^2^) was classified as ≤29.9 (normal or overweight) and ≥30.0 (obese). Current daily smoking (no/yes) was enquired in the interview. There were two categories for *leisure time physical exercise*: exercising at least once a week (active)/more seldom (passive).Those who had worked at any time during the preceding year were classified as *occupationally active* (yes/no). Exposure to physical strain in one’s most recent occupation was elicited in the home interview. *Physically strenuous work* comprised tasks such as lifting or carrying heavy objects, excavating, digging and pushing; yes/no. Psychosocial strain was measured using the Job Content Questionnaire [30]. The scales of *job demands* comprised five items (Cronbach’s alpha, α = 0.79), and *job control* nine items (α = 0.84). Participants with a high job demand level (above the median) and a low job control level (below the median) were assumed to have job strain. We substituted missing values (10.2%) with the mean of each scale.

### Statistical analyses

When all-cause sickness absence was analysed, the follow-up of each participant in different alcohol use categories began on the day they first took part in the Health 2000 Survey and ended with the award of a sickness absence benefit or pension, death, or the end of follow-up (31.12.2010), whichever occurred first. When sickness absence due to mental or musculoskeletal disorders was considered, the follow-up started on 1 Jan, 2004 because data on the diagnoses was not available until that date. To test the robustness of results we performed additional analyses that included also information of the sample based diagnoses before 2004 (presented in Table 5).

We fitted Cox proportional hazards regression models (PROC SURVIVAL) in order to examine the relationship between alcohol use categories and the occurrence of first subsequent episode of sickness absence from the beginning of follow-up for 1) all causes, 2) musculoskeletal disorders, and 3) mental disorders. We also examined, in three models, the effect of demographic factors, chronic diseases and disorders, health behavior and occupational factors at baseline, on the relationship between alcohol use and sickness absence. We tested the assumption of proportional hazards with the Kolmogorov-type supremum test, which indicated that the assumption for each model was satisfied. We took the sampling design into account in all statistical analyses. Post-stratification weights, which were calibrated using age, sex, living district and language, were applied to correct the effects of non-response. We used SAS software package (version 9.4; SAS Institute, Inc., Cary, North Carolina) and SUDAAN software (release 11) for the statistical analyses.

## Results

The mean age of participants at baseline was 43 (Table 1), and 48% were men. From 2000 to 2010, 56% of participants had at least one medically certified sickness absence spell that lasted for more than 10 days (men 51%, women 61%). The most usual causes of long-term sickness absence were musculoskeletal (41%) and mental disorders (20%).

**Table 1:** Descriptive information of alcohol use and covariates at baseline among all Finns and those with sickness absence (events) in the Health 2000 survey. All proportions are weighted

	Participants		Events 2000-2010	Events 2004-2010
			*N* = 2051	*N* = 1633
	N	%	%	%
Alcohol use as predictor				
Lifelong abstainer	227	6.0	5.5	5.3
Former drinker	498	13.5	14.2	14.6
*User, alcohol portions/week*				
Light (1 − 6 (men), 1 − 4 (women)	1406	38.6	37.6	38.2
Moderate (7 − 22 (men), 5 − 11 (women)	945	27.8	27.1	26.8
High-volume drinker (≥ 23 (men), ≥12 (women)	397	8.9	9.6	9.5
Participants with an alcohol use disorder	193	5.2	6.1	5.6
All	3666	100	100	100
Covariates				
Age, mean (SD)	42.7 (7.5)		43.4 (7.3)	43.4 (7.3)
Gender, men	1739	47.6	45.9	42.1
Marital status, single vs. married/co-habiting	851	23.3	24.1	23.5
Chronic musculoskeletal disorders ≥ 1	1004	27.5	32.3	31.4
Other chronic somatic disorders ≥ 1	1243	33.8	37.5	36.8
Common mental disorders ≥ 1	526	14.4	17.5	17.3
*Other health behaviour*				
BMI ≥ 30	639	17.6	20.5	20.9
Daily smoking, yes vs. no	996	27.3	31.0	30.3
Leisure time exercise, passive vs. active	926	25.1	26.8	26.2
*Occupational factors*				
Educational level ≤ 9 yrs.	651	17.5	20.4	18.3
Job strain, yes vs. no	525	14.4	16.3	15.9
Occupational active; yes vs. no	3378	92.4	92.9	93.7
Physically strenuous work; yes vs. no	939	25.6	30.6	29.5

Among those with no current alcohol use, it was more common to be a former drinker (14%) than a lifelong abstainer (6%) (Table 1). Most of the participants were light or moderate drinkers (66%). People who drank weekly at a hazardous level or had an alcohol use disorder comprised 15% of all participants.

Most of the lifelong abstainers, former drinkers and light drinkers were women, while moderate and heavy drinkers were usually men (Table 2). The former drinkers were younger than lifelong abstainers and current alcohol users and were more seldom occupationally active than alcohol users. Lifelong abstainers were older, had less education, had more often physically strenuous work and were more often occupationally inactive than those who used alcohol (Table 2). Furthermore, lifelong abstainers and light drinkers were less often smokers, while about a half of high-volume drinkers and participants with alcohol use disorders were current smokers.

**Table 2:** Characteristics of participants in different alcohol use categories among 30 − 55-year-old Finns in the Health 2000 survey. All proportions are weighted

	Lifelong abstainer	Former drinker	Light drinker	Moderate drinker	High-volume drinker	Participants with an alcohol use disorder	Total
	% (95% CI)^a^	% (95% CI)^a^	% (95% CI)^a^	% (95% CI)^a^	% (95% CI)^a^	% (95% CI)^a^	% (95% CI)^a^
Men	29.6 (24.6 − 30.6)	30.1 (28.0 − 32.2)	38.7 (37.4 − 40.0)	62.1 (60.6 − 63.6)	60.8 (58.1 − 63.5)	79.8 (58.1 − 63.5)	47.5 (46.7 − 48.3)
Age > 45 yrs.	48.0 (44.7 − 51.3)	34.7 (32.6 − 36.8)	37.4 (36.1 − 38.7)	39.2 (37.7 − 40.7)	43.8 (41.1 − 46.5)	30.1 (26.8 − 33.4)	38.4 (37.6 − 39.2)
Educational level ≤ 9 yrs.	25.3 (22.4 − 28.2)	20.1 (18.3 − 21.9)	14.2 (13.3 − 15.1)	19.6 (18.4 − 20.8)	19.5 (17.3 − 21.7)	16.1 (13.5 − 18.7)	17.7 (17.1 − 18.3)
Physically strenuous work; yes	36.4 (33.2 − 39.6)	27.7 (25.7 − 29.7)	22.4 (21.3 − 23.5)	25.6 (24.2 − 26.9)	26.4 (24.0 − 28.9)	30.1 (26.2 − 33.4)	25.6 (24.9 − 26.3)
Occupational active; yes	88.0 (85.8 − 90.2)	87.4 (85.9 − 88.9)	93.7 (93.1 − 94.4)	95.5 (94.8 − 96.1)	90.0 (88.3 − 91.7)	85.0 (82.4 − 87.6)	92.2 (91.8 − 92.6)
Current smoker; yes	10.7 (8.7 − 12.7)	27.5 (25.5 − 29.5)	19.3 (18.2 − 20.3)	30.8 (29.3 − 32.3)	46.8 (44.0 − 49.6)	50.3 (46.7 − 53.9)	27.2 (26.4 − 27.9)

^a^Confidence Interval

### Alcohol use and all-cause sickness absence

Lifelong abstainers, former drinkers or moderate or high volume alcohol users, were not at an increased risk for a long-term sickness absence due to any cause, when compared to light drinkers (Table 3). However, the age-adjusted HR of participants with alcohol use disorder was 1.45 (95% CI = 1.19 − 1.76), decreasing to 1.35 (95% CI = 1.11 − 1.64) when also demographic, other lifestyle-related and occupational factors were added among the covariates, and further to 1.27 (95% CI = 1.04 − 1.54) when also somatic and mental disorders were accounted for (Table 3, Model 3).

**Table 3:** Alcohol use among Finns in the Health 2000 Survey in association with the occurrence of first subsequent sickness absence lasting over 10 days in 2000 − 2010, certified for any causes. Follow-up started in 2000-2001

Adjusted for following baseline characteristics:		Model 1 Age		Model 2 Age, marital status, education, other health behavior and occupational factors		Model 3 All aforementioned, musculoskeletal and mental disorders and other diseases/disorders	
Reference = 1	Events	HR^a^	95% CI^B^	HR^a^	95% CI^b^	HR^a^	95% CI^b^
*For any cause (N = 2051)*							
Lifelong abstainer	116	0.86	0.71, 1.05	0.83	0.68, 1.02	0.82	0.67, 1.00
Former drinker	297	1.14	1.00, 1.30	1.04	0.91, 1.20	1.07	0.93, 1.22
Light drinker	777	1.00	Reference	1.00	Reference	1.00	Reference
Moderate drinker	509	1.04	0.93,1.17	1.00	0.89, 1.11	0.94	0.87, 1.11
High-volume drinker	230	1.15	0.98, 1.35	1.05	0.90, 1.24	1.05	0.89, 1.24
Participants with alcohol use disorder	122	1.45	1.19, 1.76	1.35	1.11, 1.65	1.27	1.04, 1.54

^a^Hazard ratio

^b^95% Confidence interval

### Alcohol use and sickness absence due to mental and musculoskeletal disorders

Lifelong abstainers’ HRs for sickness absence due to mental disorders did not differ from that of light drinkers but the age-adjusted HR for former drinkers was 1.61; 95% CI = 1.18 − 2.19 (Table 4, Model 1). With all covariates in the model, former drinkers were still at an increased risk (HR 1.57; 95% CI = 1.15 − 2.15) (Table 3, Model 3).

**Table 4:** Alcohol use among Finns in the Health 2000 Survey in association with the occurrence of first subsequent sickness absence lasting over 10 days in 2004 − 2010, certified for mental and musculoskeletal disorders

Adjusted for following baseline characteristics:		Model 1 Age		Model 2 Age, marital status, education, other health behavior and occupational factors		Model 3 All aforementioned, musculoskeletal and mental disorders and other diseases/disorders	
Reference = 1	Events	HR^a^	95% CI^b^	HR^a^	95% CI^b^	HR^a^	95% CI^b^
*For mental disorders (N = 336)*							
Lifelong abstainer	19	1.06	0.65, 1.72	1.09	0.67, 1.79	1.10	0.67, 1.80
Former drinker	63	1.61	1.18, 2.19	1.56	1.14, 2.14	1.57	1.15, 2.15
Light drinker	109	1.00	Reference	1.00	Reference	1.00	Reference
Moderate drinker	82	1.28	0.95, 1.71	1.23	0.92, 1.65	1.19	0.89, 1.59
High-volume drinker	35	1.70	1.16, 2.50	1.57	1.06, 2.33	1.52	1.03, 2.25
Participants with alcohol use disorder	28	3.05	1.98, 4.68	2.81	1.82, 4.35	2.16	1.39, 3.35
*For musculoskeletal disorders (N = 737)*							
Lifelong abstainer	44	0.89	0.65, 1.23	0.87	0.63, 1.19	0.89	0.64, 1.23
Former drinker	127	1.29	1.05, 1.59	0.18	0.97, 1.48	1.21	0.98, 1.49
Light drinker	288	1.00	Reference	1.00	Reference	1.00	Reference
Moderate drinker	190	1.00	0.84, 1.20	0.95	0.79, 1.15	0.94	0.78, 1.14
High-volume drinker	49	0.77	0.57, 1.04	0.74	0.55, 1.01	0.74	0.54, 1.00
Participants with alcohol use disorder	39	1.26	0.90, 1.77	1.28	0.91, 1.63	1.23	0.87, 1.74

^aHazard ratio^

^b 95% Confidence interval^

The HR for subsequent sickness absence due to mental disorders was 1.52 for high-volume drinkers (95% CI = 1.03 − 2.25) and 2.16 (95% CI = 1.39 − 3.35) for those with an alcohol use disorder, when all covariates were accounted for (Table 4, Model 3). Moderate drinking was not associated with sickness absence due to mental disorders.

The risk of sickness absence due to mental disorders among participants with an alcohol use disorder was clearly increased: the age-adjusted HR was 3.05 (95% CI = 1.98 − 4.68; Model 1), decreasing to 2.16 (95% CI = 1.39-3.35) when all covariates were included in the model (3). Somatic and mental disorders at baseline explained 23% of the total decrease in HRs (Models 2 and 3). Among high-volume drinkers, adjustments explained only 11% of the decrease of HRs from the age-adjusted model to full model (Models 1 and 3).

Former drinkers were at an increased age-adjusted risk of long-term sickness absence due to musculoskeletal disorders (HR = 1.29; 95% CI = 1.05 − 1.59) (Table 4, Model 1) compared to light drinkers. However, accounting for all covariates, the HR decreased to statistically non-significant level (HR = 1.21; 95% CI = 0.98 − 1.49). No other associations of alcohol use with sickness absence due to musculoskeletal disorders were found.

## Discussion

In this nationally representative prospective study, alcohol use disorder was associated with subsequent long-term sickness absence due to all causes and due to mental disorders in particular. High-volume drinking and former drinking also predicted long-term sickness absence due to mental disorders, but the risk of lifelong abstainers did not differ from that of light drinkers when somatic and mental disorders as well as demographic, lifestyle-related and occupational factors at baseline were accounted for. No associations due to musculoskeletal disorders were found.

### Interpretation of results

A review by Schou et al. [4] suggests an overall positive association between alcohol use and sickness absence but this seems to be somewhat stronger for short-term than long-term absences. However, only four of the 28 included articles in the review had a longitudinal design. Our results were based on sickness absence periods that lasted for over 10 days, and thus do not necessarily apply to short-term sickness absence spells.

In a recent study of French national electricity and gas company workers was found that even moderate daily alcohol consumption was associated with an increased risk of sickness absence, especially of long duration (> 28 days) [15]. The authors also found a relationship between high alcohol consumption and long sickness absence periods due to mental and musculoskeletal disorders among men.

Our study results are in line with previous findings that alcohol use disorders are related to many somatic [31] and psychiatric conditions [32]. We also observed that baseline somatic and mental disorders explained about a quarter of the association between alcohol use disorders and subsequent long-term sickness absence due to mental disorders.

However, our study did not confirm the findings of some previous prospective studies [9–11] that abstinence from alcohol increases sickness absence. Our study extends previous evidence by distinguishing between lifelong abstinence and former drinking and showing for the first time that only former drinking is associated with the risk of sickness absence. Thus, in previous studies, the risk between non-drinking and sickness absence could have reflected that for former drinkers only. Our results further showed that former drinkers in particular had more often long-term sickness absence due to mental disorders compared to light drinkers. This is in line with previous observations that the health of former alcohol users is worse than that of light or moderate drinkers [33], and former users had also reported ceasing alcohol consumption for health reasons [34]. Such health problems as hypertension, diabetes and anxiety were reasons to reduce or stop alcohol consumption [35]. In a study concerning mental health among people aged 65 or over, lifelong abstaining was associated with better lifetime mental history while ex-drinkers had a worse past-year mental health status compared with those who drank alcohol but were not binge-drinkers [36].

There is a lack of information about the characteristics of people who have quit drinking. Our results showed that former drinkers were more often women and younger than 45 years and less often occupationally active than alcohol users. The results did not change even when we removed the 49 women who were pregnant when the survey was carried out. Former drinkers did not differ from average according to their educational level, physical strenuousness of most recent work or smoking. It is then possible that they have quit drinking because of mental symptoms or medication. A previous study found that former drinkers achieved worse scores on several measures of well-being and mental health compared to lifelong abstainers [37].

Many studies have shown that excessive alcohol use doubles the risk of depressive symptoms and vice versa, but the primary direction of associations is still unclear [38, 39]. Based on studies of male twins, Davis and colleagues [40] suggest that reciprocal causation exists between alcohol dependence and major depressive disorder (MDD), whereby MDD increases the risk for alcohol dependence and alcohol dependence increases the risk of MDD. It has been reported that especially binge drinking, measured as drunkenness, intoxications, hangovers and alcohol-induced pass-outs, predicts future depressive symptoms [41].

It is possible that some people with mental health problems try to raise their blue mood by alcohol. In a nationally representative study based on retrospective data, common affective and anxiety disorders increased the risk of alcohol misuse and alcohol dependence [42]. Also in a case-control study alcohol dependence was more prevalent among anxious or depressed people and it was more likely to be a secondary than primary disorder also among women [43]. A cross-sectional study of a population-based sample showed consistent associations between early debut of alcohol use and more symptoms of mental health problems [44]. Anyway, regardless of the temporal sequencing of excessive alcohol use and mental disorders, it is essential in health care to identify the potential co-occurrence of these disorders.

### Methodological considerations

Our study had a prospective design and nationally representative data of the Finnish population with register-based information on medically certified and compensated sickness absence. The participation rate was high, and the information on alcohol use was diverse with little missing data. Most of the items in the questionnaires, interviews and health examination were selected on the basis of standard recommendations or nationally established practice [22]. We used the standardized CIDI interview to assess alcohol use disorders and common mental disorders according to DSM-IV diagnostic criteria. Chronic mental and somatic disorders were diagnosed by specially trained physicians who followed predetermined clinical protocol.

One limitation was that the determinants were measured only once and we had no information on the possible changes, especially in alcohol use, that may increase or decrease over time [45]. Another weakness involves the sample-based information in diagnoses of sickness absence spells from 2000 to 2003. Therefore, we started the follow-up in diagnosis-based analyses only from 2004 with the complete information and thereafter made additional analyses with the sample-based data starting the follow-up from 2000 (Appendix), for to control that the results were similar.

## Conclusion

Excessive alcohol use, clinical alcohol use disorders and former drinking were associated with an increased risk of long-term sickness absence but lifelong abstinence was not. Alcohol use disorder, in particular, but also high-volume drinking and former drinking were predictive of sickness absence due to mental disorders. It is important within health care to identify people with problematic alcohol use and to map and support their mental wellbeing when attempting to prevent long-term sickness absence.

## Appendix

**Table 5:** Alcohol use among Finns in the Health 2000 Survey in association with the occurrence of first subsequent sickness absence lasting over 10 days in 2000−2010, certified for musculoskeletal- and mental disorders. Diagnoses in 2000-2003 were based on a random sample

Adjusted for following baseline characteristics:		Model 1 Age		Model 2 Age, marital status, education, other health behavior and occupational factors		Model 3 All aforementioned, musculoskeletal and mental disorders and other diseases/disorders	
Reference=1	Events	HR^a^	95% CI^b^	HR^a^	95% CI^b^	HR^a^	95% CI^b^
*For mental disorders (N=416)*							
Lifelong abstainer	29	1.34	0.89, 2.01	1.30	0.86, 1.95	1.28	0.85, 1.92
Former drinker	75	1.63	1.23, 2.17	1.51	1.13, 2.02	1.50	1.16, 2.07
Light drinker	127	1.00	Reference	1.00	Reference	1.00	Reference
Moderate drinker	94	1.35	1.04, 1.76	1.28	0.98, 1.68	1.25	0.96, 1.63
High-volume drinker	51	1.76	1.24, 2.49	1.55	1.09, 2.21	1.52	1.07, 2.17
Participants with alcohol use disorder	40	3.65	2.53, 5.28	3.16	2.17, 4.60	2.37	1.62, 3.48
*For musculoskeletal disorders (N=841)*							
Lifelong abstainer	49	0.85	0.63, 1.15	0.80	0.59, 1.08	0.75	0.55, 1.02
Former drinker	143	1.31	1.07, 1.59	1.16	0.95, 1.42	1.19	0.98, 1.45
Light drinker	328	1.00	Reference	1.00	Reference	1.00	Reference
Moderate drinker	207	1.00	0.84, 1.20	0.96	0.80, 1.14	0.94	0.79, 1.12
High-volume drinker	72	0.80	0.60, 1.05	0.75	0.56, 1.00	0.74	0.56, 1.01
Participants with alcohol use disorder	42	1.24	0.89, 1.71	1.17	0.84, 1.63	1.12	0.81, 1.57

^a Hazard ratio^

^b 95 % Confidence interval^

## Abbreviations

BMI: Body mass index
CI: Confidence interval
CIDI: Composite International Diagnostic Interview,
DSM-IV: Diagnostic and Statistical Manual of Mental Disorders
HR: Hazard ratio
ICD: International classification of diseases
MDD: Major depressive disorder
QF: The quantity-frequency method
SD: Standard deviation
